# Mapping geographical inequalities of incomplete immunization in Ethiopia: a spatial with multilevel analysis

**DOI:** 10.3389/fpubh.2024.1339539

**Published:** 2024-06-07

**Authors:** Berihun Bantie, Natnael Atnafu Gebeyehu, Getachew Asmare Adella, Gizachew Ambaw Kassie, Misganaw Asmamaw Mengstie, Endeshaw Chekol Abebe, Mohammed Abdu Seid, Molalegn Mesele Gesese, Kirubel Dagnaw Tegegne, Melkamu Aderajew Zemene, Denekew Tenaw Anley, Anteneh Mengist Dessie, Sefineh Fenta Feleke, Tadesse Asmamaw Dejenie, Ermias Sisay Chanie, Solomon Demis Kebede, Wubet Alebachew Bayih, Natnael Moges, Yenealem Solomon Kebede

**Affiliations:** ^1^Department of Comprehensive Nursing, College of Health Sciences, Debre Tabor University, Debre Tabor, Ethiopia; ^2^Department of Midwifery, College of Medicine and Health Science, Wolaita Sodo University, Wolaita, Ethiopia; ^3^Department of Reproductive Health and Nutrition, School of Public Health, Woliata Sodo University, Sodo, Ethiopia; ^4^Department of Epidemiology and Biostatistics, School of Public Health, Woliata Sodo University, Sodo, Ethiopia; ^5^Department of Biochemistry, College of Health Sciences, Debre Tabor University, Debre Tabor, Ethiopia; ^6^Unit of Physiology, Department of Biomedical Science, College of Health Science, Debre Tabor University, Debre Tabor, Ethiopia; ^7^Department of Nursing, College of Medicine and Health Science, Wollo University, Dessie, Ethiopia; ^8^Department of Public Health, College of Health Sciences, Debre Tabor University, Debre Tabor, Ethiopia; ^9^Department of Public Health, College of Health Sciences, Woldia University, Woldia, Ethiopia; ^10^Department of Medical Biochemistry, College of Medicine and Health Sciences, University of Gondar, Gondar, Ethiopia; ^11^Department of Pediatrics and Child Health Nursing, College of Health Sciences, Debre Tabor University, Debre Tabor, Ethiopia; ^12^Department of Medical Laboratory Science, College of Health Sciences, Debre Tabor University, Debre Tabor, Ethiopia

**Keywords:** geographical inequalities, incomplete immunization, children, Ethiopia, spatial analysis

## Abstract

**Background:**

Immunization is one of the most cost-effective interventions, averting 3.5–5 million deaths every year worldwide. However, incomplete immunization remains a major public health concern, particularly in Ethiopia. The objective of this study is to investigate the geographical inequalities and determinants of incomplete immunization in Ethiopia.

**Methods:**

A secondary analysis of the mini-Ethiopian Demographic Health Survey (EDHS 2019) was performed, utilizing a weighted sample of 3,865 children aged 12–23 months. A spatial auto-correlation (Global Moran's I) statistic was computed using ArcGIS version 10.7.1 to assess the geographical distribution of incomplete immunization. Hot-spot (areas with a high proportion of incomplete immunization), and cold spot areas were identified through Getis-Ord Gi^*^ hot spot analysis. Additionally, a Bernoulli probability-based spatial scan statistics was conducted in SaTScan version 9.6 software to determine purely statistically significant clusters of incomplete immunization. Finally, a multilevel fixed-effects logistic regression model was employed to identify factors determining the status of incomplete immunization.

**Results:**

Overall, in Ethiopia, more than half (54%, 95% CI: 48–58%) of children aged 12–23 months were not fully immunized. The spatial analysis revealed that the distribution of incomplete immunization was highly clustered in certain areas of Ethiopia (Z-score value = 8.379419, *p*-value < 0.001). Hotspot areas of incomplete immunization were observed in the Afar, Somali, and southwestern parts of Ethiopia. The SaTScan spatial analysis detected a total of 55 statistically significant clusters of incomplete immunization, with the primary SaTScan cluster found in the Afar region (zones 1, 3, and 4), and the most likely secondary clusters detected in Jarar, Doola, Korahe, Shabelle, Nogob, and Afdar administrative zones of the Somali region of Ethiopia. Indeed, in the multilevel mixed-effect logistic regression analysis, the respondent's age (AOR: 0.92; 95% CI: 0.86–0.98), residence (AOR: 3.11, 95% CI: 1.36–7.14), living in a pastoralist region (AOR: 3.41; 95% CI: 1.29–9.00), educational status (AOR: 0.26; 95% CI: 0.08–0.88), place of delivery (AOR: 2.44; 95% CI: 1.15–5.16), and having PNC utilization status (AOR: 2.70; 95% CI: 1.4–5.29) were identified as significant predictors of incomplete immunization.

**Conclusion and recommendation:**

In Ethiopia, incomplete immunization is not randomly distributed. Various factors at both individual and community levels significantly influence childhood immunization status in the country. It is crucial to reduce disparities in socio-demographic status through enhanced collaboration across multiple sectors and by bolstering the utilization of maternal health care services. This requires concerted efforts from stakeholders.

## 1 Introduction

Immunization is one of the most effective public health interventions, giving every child the opportunity to grow up healthy and reach their full potential (1, 2). The expanded program on immunization (EPI) was launched in 1974 with the objective to ensure that all children, in all countries, benefited from life-saving vaccines (3). Nowadays, more than 20 life-threatening diseases can be prevented by these vaccines, helping people of all ages live longer, healthier lives. In this context, about 3.5–5 million deaths every year worldwide were prevented by vaccination, and it is also predicted that 51 million deaths will be prevented through immunization between 2021 and 2030 (2, 4, 5). On the other hand, by the end of 2021, one in five children globally do not have access to essential immunizations (4). Furthermore, about 25 million children under the age of 1 year did not receive basic vaccines (3, 4). The burden of incomplete immunization, however, is not evenly distributed worldwide, with the highest figures observed in low- and middle-income countries, particularly in Africa and South-East Asia (4, 6).

In Ethiopia, the Expanded Program on Immunization (EPI) commenced in 1980, initially covering six antigens: BCG, Diphtheria (D), Pertussis (P), Tetanus (T), Polio, and Measles (7). Since then, the program has been expanded and now features a comprehensive vaccination schedule, including the administration of the Bacillus Calmette-Guérin (BCG) and oral polio vaccine (OPV) at birth. This is followed by three doses of pentavalent vaccine, PCV, and OPV at 6, 10, and 14 weeks, as well as two doses of rotavirus vaccine at 10 and 14 weeks, and a measles vaccine at 9 months (7, 8). In line with this, according to WHO, children are considered fully immunized if they have been vaccinated against tuberculosis (BCG), received three doses of the DPT-HepB-Hib (pentavalent) vaccine, three doses of the PCV, two doses of the rotavirus vaccine, and vaccinations against polio and measles (1, 2). On the contrary, Ethiopia is among the top ten countries in the world with the largest number of children receiving zero-dose vaccination, ranking second next to Nigeria in Africa (4, 9). Consequently, vaccine-preventable diseases, namely pneumonia (17%), diarrhea (8%), and measles (4%), are the leading causes of death among children's under the age of five in Ethiopia (8). In response to this pressing issue, the Ethiopian government designed and implemented various strategies. These included mobilizing volunteers, health extension workers, and healthcare facilities to expand immunization services, with the aim of substantially reducing morbidity and mortality among children under five from vaccine-preventable diseases (8, 10). Following to these efforts, there has been a slight improvement in immunization coverage (8, 11), with a noteworthy reduction in under-five mortality rates (11, 12). However, recent reports indicate that only 44% of children aged 12–23 months in Ethiopia have received all essential vaccinations or are fully immunized (11). The WHO immunization agenda for 2030, on the other hand, sets a target of 90% national full or complete vaccination coverage, indicating that Ethiopia is too far from meeting this target (8, 13). Various interconnected and interrelated individual, community, and service delivery-related factors affect the childhood immunization level in the country. Existing literature has primarily shown that individual-level factors, including mothers' educational status, antenatal care follow-up, place of delivery, postnatal care, residence, media exposure, and wealth level, play a significant role in determining the level of childhood immunization (10, 14–20). In contrast, the influence of community-level factors on incomplete immunization has not been adequately investigated in Ethiopia. Previous studies have also highlighted variations in incomplete immunization coverage across different regions and districts in Ethiopia (11, 15, 16). However, data on the spatial distribution of incomplete immunization are also very limited in Ethiopia. Mapping geographical inequalities of incomplete immunization using spatial analysis in Ethiopia is therefore essential for identifying hotspot and cold spot areas, informing the mobilization of resources, and designing targeted interventions to improve vaccination coverage and reduce disparities (21, 22). Hence, the main aim of this study is to identify the high- and low-risk regions as well as the individual and community-level factors of incomplete immunization using the latest EDHS (Mini-EDHS 2019) survey.

## 2 Methods

### 2.1 Study design, setting, and period

A secondary analysis of the 2019 mini-Ethiopian Demographic and Health Survey (mini-EDHS), conducted from March 21, 2019, to June 28, 2019, was utilized to investigate spatial variation and determinants of incomplete immunization. This survey was a nationwide population-based cross-sectional study conducted in all regions of Ethiopia by the Central Statistical Agency in collaboration with the Federal Ministry of Health (FMoH) and the Ethiopian Public Health Institute (EPHI) (11). Ethiopia is the second-most populous country in Africa, with a total population of over 117 million (23). The nation is administratively divided into nine regions and two town administrations, comprising 68 zones, 817 districts, and 16,253 kebeles (the smallest administrative unit in the country). Kebeles are further subdivided into census Enumeration Areas (EAs) or clusters, which serve as the sampling frame for surveys.

### 2.2 Data sources and populations

This study used 2019 mini Ethiopia Demographic Health Survey (EDHS) data, which can be obtained from the DHS database: http://www.dhsprogram.com. Information on vaccination coverage was collected in three ways in the 2019 EMDHS: from written vaccination records, including the infant immunization card and other health cards; from mothers' verbal reports; and from health facility records. All children age 12–23 months in were the source population, whereas all children's aged 12–23 months in the selected EAs were the study populations.

### 2.3 Sample size determination and sampling technique

The final study participants were chosen using a two-stage stratified cluster sampling technique. As described in detail in the EDHS 2019 report (11), in the first stage, 305 enumeration areas (EAs), consisting of 93 urban and 212 rural areas, were selected with probability proportional to their size based on the 2019 Ethiopian Population and Housing Census (EPHC) frame. In the second stage of selection, a fixed number of 30 households per cluster were chosen with an equal probability of systematic selection from the household listing newly created for the survey. In the selected households, a total of 8,855 women of reproductive age (aged 15–49) were interviewed, and 5,753 under-five children were found. Finally, after recruiting the most recent births in households with two or more children aged 12–23 months and excluding incomplete vaccination status records, a total of 1,069 children (weighted sample of 1,097) were included in the final analysis. Due to the unequal distribution of samples among various regions, where regions with small populations are oversampled and vice versa, as well as potential variations in response rates within the Ethiopian Demographic and Health Survey (EDHS) of 2019, it is imperative to utilize sampling weights for all analyses to ensure accurate representation. The detailed sampling procedure was included in the EDHS report (11).

### 2.4 Variables of the study

#### 2.4.1 Dependent/outcome variable

The outcome variable in this study was the presence of incomplete immunization. It was categorized into binary (yes/no) categories.

#### 2.4.2 Explanatory/independent variables

The factors influencing incomplete immunization status in Ethiopia were broadly categorized into individual and community-level factors. Individual-level variables included age of the mother, educational status of women and husband, marital status, household wealth index, receipt of counseling and education, sex of the head of the household (HH), receipt of Antenatal care (ANC), place of birth, birth order, sex of the child, and receipt of postnatal care. Community-level factors included region, residence, community poverty level, and community education status. Community-level factors were obtained by aggregating individual-level characteristics at the community (cluster) level. The aggregation of variables was categorized as high or low based on the distribution of the data (using either median or mean).

### 2.5 Operational definitions

According to the World Health Organization, a child is considered fully (completely) immunized, when he or she has received one dose of BCG, three doses of pentavalent (Penta-vaccine), pneumococcal conjugate (PCV), oral polio vaccines (OPV), two doses of rotavirus, and one dose of measles vaccine (24). Hence, if the child who started vaccination missed at least one dose of vaccination (one dose of BCG, three doses of polio from four doses, three doses of pentavalent, and one dose of measles) at any time between 0 and 23 months, he/she was categorized as having an incomplete immunization status. Community poverty status is defined as the proportion of households in the poorer or poorest quintile of each cluster (25). Community women's educational level is also defined as the proportion of women who have completed at least a primary level of education in each cluster (25).

### 2.6 Data processing and analysis

After the extraction of relevant data, further cleaning, coding, and analysis were done using Microsoft Excel 2013 and STATA V.16 software. A weighted sample was used to ensure the representativeness of the survey by reducing sampling variability (11). Frequency with percentage, mean with standard deviation, median with interquartile range were used to summarize data.

Considering the spatially varying relationships of variables in clustered data, Ordinary Linear Regression (OLR) and Geographically Weighted Regression (GWR) were considered to identify predictors of incomplete immunization (26, 27). However, the assumptions of OLS were not satisfied. The model residuals were not normally distributed (Shapiro-Wilk test with *p* < 0.01) and revealed a spatial pattern with a spatial autocorrelation result of 0.84317 Moran's Index with a *p*-value < 0.05. Additionally, the Koenker statistic in GWR was not statistically significant (*p* > 0.05). These findings suggest that the relationship between each explanatory variable and incomplete immunization did not vary (stationary) across geographical areas. Instead, a multilevel analysis, which effectively handles the dependence of observations within clusters in hierarchical data, would be preferable. Thus, a multilevel mixed-effect logistic regression model was employed, particularly after observing an intraclass correlation value (ICC > 5%) (28–30). Initially, bi-variable multilevel mixed-effect logistic regression models were computed, wherein all variables with a *p*-value ≤ 0.25 were included in the final multivariable logistic regression model. Following the selection of variables for the multivariable analysis, four models were fitted sequentially: the null model (a model without explanatory variables is used to test random variability in the intercept and to estimate the intra-class correlation coefficient); model 1 (containing only individual-level factors); model 2 (containing only community-level factors); and model 3 (which comprises both individual and community-level factors). The log likelihood ratio (LLR), device, and Akaike Information Criterion (AIC) were used to compare and select the best model. The best model was determined by having the highest log likelihood, the lowest deviance and lowest AIC. Finally, adjusted odds ratios (AOR) with 95% confidence intervals (CI) were reported in the multivariable, multilevel mixed- effect logistic regression model and statistical significance was declared at a *p*-value of < 0.05. A pseudo-linear regression analysis was used to test for the presence of multi-collinearity among independent variables using a variance inflation factor (VIF > 10).

#### 2.6.1 Random effect (community level variation) analysis

The random effect (the amount of community level variation) of incomplete immunization across clusters was assessed using ICC, proportional change in variance (PCV), and the median odds ratio (MOR) (29). The MOR was used to determine the heterogeneity of incomplete immunization between clusters (the second-level variation) by comparing two people from two randomly selected clusters. Proportional change in variance (PCV) is used to assess the total variation attributed to individual and community level factors in the multilevel model as compared to the null model.

#### 2.6.2 Spatial distribution analysis

ArcGIS 10.7.1 software was used to investigate the spatial distribution and identify high and low-risk areas incomplete immunization in Ethiopia. The Global Moran's I (spatial auto-correlation) test statistic was employed to assess whether incomplete immunization is dispersed, clustered or random distributed (31). The Moran's I values near −1 denote a dispersed pattern, while values near 0 indicate a random pattern and those near +1 signifies clustering distributional pattern of incomplete immunization in Ethiopia The maximum distance at which incomplete immunization become more prevalent was determined through incremental spatial auto-correlation test.

After assessing the overall spatial autocorrelation status of incomplete immunization, a local Moran hot spot analysis was conducted to identify spatial clusters with high values (hot-spot areas) and low values (cold-spot areas). Hot-spot areas indicate clusters with a high proportion of incomplete immunization, whereas cold spots signify a low proportion of incomplete immunization (32). In addition, a spatial interpolation using ordinary Kriging spatial interpolation m technique was also computed to predict incomplete immunization in unsampled areas of the country based on sampled Enumeration Areas (EAs).

Consistently, in the presence of clustering pattern, a Bernoulli probability-based model of spatial scan statistics was computed using SaTScan version 9.6 software to determine purely statistically significant spatial clusters (33). The scanning window, moving across the study area, considered children with incomplete immunization as “cases” and those without as “controls”. The model required data for cases, controls, and geographic coordinates. The default maximum spatial cluster size of 50% of the population was applied as an upper limit, enabling detection of both small and large clusters. The likelihood ratio test statistic was used for each potential cluster to determine if observed cases were significantly higher than expected cases within the cluster. Primary, secondary, and tertiary clusters were then identified and ranked based on likelihood ratio tests from 999 Monte Carlo replications. Finally, a relative risk (RR) was reported for each potential cluster, indicating the likelihood of incomplete immunization among children within the spatial window compared to children outside of the spatial window.

### 2.7 Ethical considerations

This study used secondary (2019 EDHS data), so informed consent was not required. Instead, permission to get access to download DHS data was obtained from the major DHS data archivist through a reasonable request using the link http://www.dhsprogram.com. In the DHS data, there are no names of individuals or household addresses. The information retrieved was only used for statistical reporting and analysis of our registered research.

## 3 Results

### 3.1 Individual and community level socio-demographic variables

A total of 1,097 children ages 6–23 months were included in the final analysis of this study. More than half (2,026, 52%) of the children were male. In terms of respondent characteristics, the mean SD of the mother's age was 28 ± 6.7 years. Indeed, the majorities of respondents (51%) lived in rural areas (73.56%) and married (93.77%). Approximately half (46.7%) and 40.31% of the community had low educational status and low wealth levels, respectively (Table 1).

**Table 1:** Socio- demographic characteristics result of respondents of incomplete immunization in Ethiopia, using EDHS 2019 survey.

**Variables (*N* = 1,097)**	**Category**	**Incomplete immunization**		**Chi-square *p*-value**	**Total weighted frequency**	**%**
		**Yes (*****N*** = **587)**	**No (510)**			
Head of the HHs	Male	507	442	0.942	949	86.5%
	Female	80	68		148	13.5%
Age category • Mean = 28 (IQR ± 6.6years)	Age 15–24	187	134	0.01	321	29.3%
	Age 25–34	295	238		533	48%
	Age 35 and above	105	138		243	22.2%
	Mean = 28 (IQR ± 6.6years)					
Marital status	Married/in union	551	486	0.905	1037	94.5%
	Not-married	36	24		60	5.5%
Educational status of the respondent	No formal education	316	182	0.001	498	45.5%
	Primary education	219	227		445	40.5%
	Secondary	33	60		93	8.5
	Tertiary and above	19	40		60	5.5
Region	Tigray	28	54	0.001	82	7.4%
	Afar	12	3		15	1.3%
	Amhara	87	153		240	21.8%
	Oromia	277	154		431	39.3%
	Somali	44	18		62	5.7%
	Benishangul	4	7		11	1.1%
	SNNPR	123	82		205	18.7%
	Gambela	2	2		4	0.4 %
	Harari	1	1		2	0.2
	Addis Abeba	5	31		36	3.3
	Dire Dewa	3	4		7	0.6
Region_category	Urban setting	10	36.	0.001	46	4.2%
	Agrarian	515	443		958	87.3
	Pastoralist	62	31		93	9.0%
Residence	Urban	127	205	0.001	332	30.3%
	Rural	460	305		765	73.5%
Wealth index	Poor	285	184	0.001	469	42.8%
	Middle	110	72		182	16.5%
	Rich	192	253		446	40.6%
Community poverty level	Poor	899	659	0.001	1,558	40.3%
	Rich	255	2,320		2,575	59.7%
Community education Status	Low	997	808	0.001	1,805	47.0%
	High	960	1,099		2,060	53.0%

IQR, inter-quartile range; HH, households.

### 3.2 Maternal and child health characteristics

In this study, more than two-thirds (5,018, or 70%) of the mothers gave birth at home. Additionally, only 60.8 and 40.9% of mothers with under-five children booked ANC follow-up and took IFA tablets during pregnancy, respectively. From the total recent births, 3,730 (52%) were males, and about 2,900 (40.5%) had normal birth weight. Regarding postnatal care services, only 422 (6.0%) of the study participants received early postnatal services within 2 days of discharge from a health facility (Table 2).

**Table 2:** Maternal and child health characteristics result of respondents of incomplete immunization using EDHS 2019 survey, Ethiopia.

**Variables (*N* = 7,168)**	**Category**	**Incomplete immunization**		**Chi-square *p*-value**	**Weighted frequency**	**%**
		**Yes (*****N*** = **587)**	**No (510)**			
ANC visit	No	202	92	0.001	294	26.8%
	Yes	385	418		804	73.2%
IFA intake status	No	303	154	0.001	457	41.7%
	Yes	284	356		640	58.3%
Received_counseling	No	154	97	0.01	251	31.3%
	Yes	231	321		552	68.7%
Delivery place	Health institution	367	183	0.001	550	50.2%
	Home	220	327		547	48.8%
Birth order	1st	129	122	0.01	251	23 %
	2^nd^-3rd	182	184		366	33%
	Four and above	276	204		480	44%
Sex of the neonate	Male	292	265	0.589	557	52.4%
	Female	295	245		540	47.6%
PNC visit	No	544	432.	0.01	977.	89 %
	Yes	43	77		120	11%

Others- Partner/husband, mother in law, father in law, someone else: IFA, iron folic acid intake.

### 3.3 Burdens of incomplete immunization in Ethiopia

In Ethiopia, more than half (54%, 95% CI: 48–58%) of children aged 12–23 months were not fully immunized. Indeed, the magnitude of incomplete immunization is highly varied across regions. The Afar (78%) and Somali regions (70.6%) have the highest prevalence rates, whereas Addis Abeba (15.18%) has the lowest burden.

### 3.4 Spatial distribution of incomplete immunization in Ethiopia

**Figure 1:**
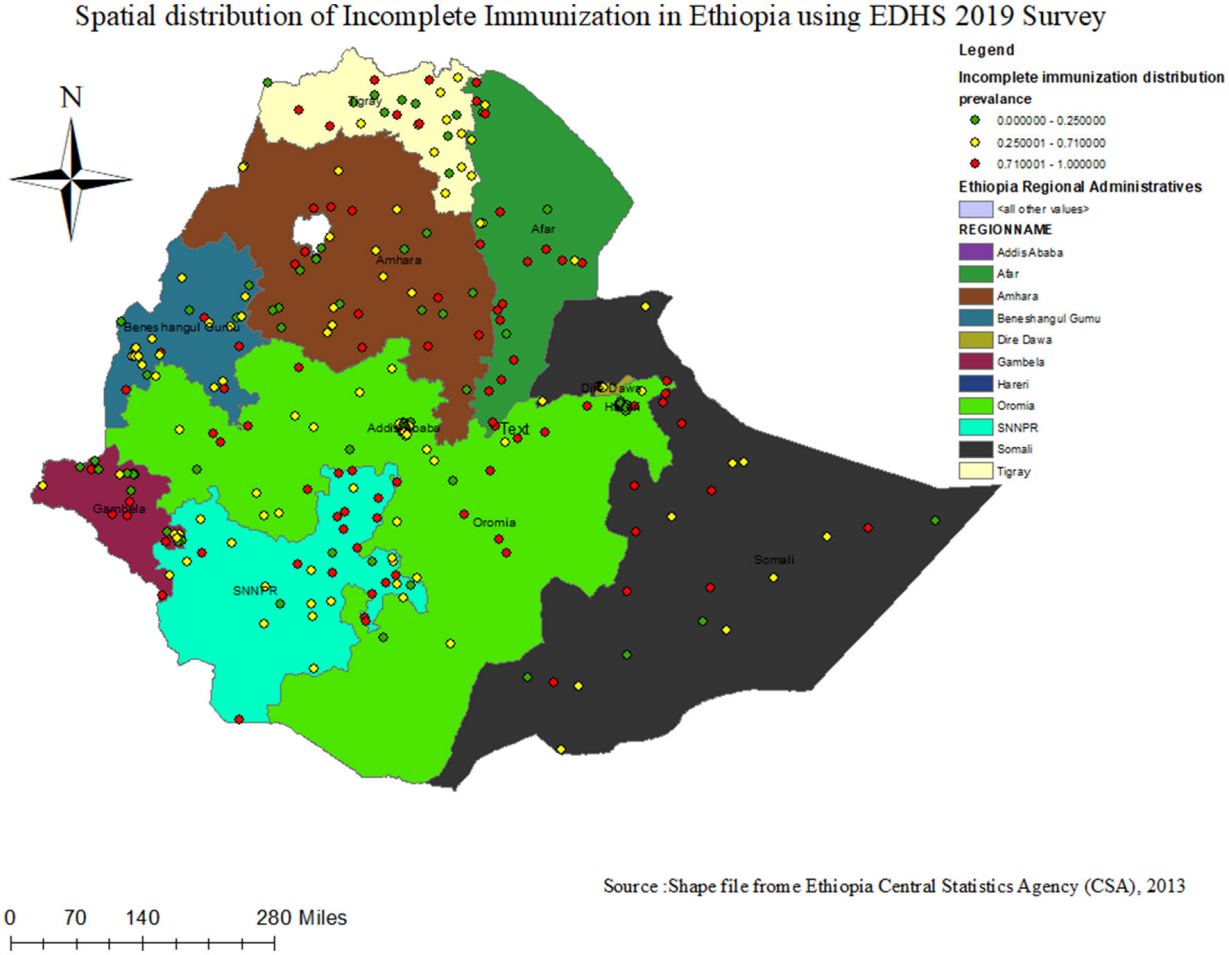
Spatial distribution status of incomplete Immunization among children 12–23 months in Ethiopia using EDHS 2019 survey.

As indicated in the figure below 2, a high proportion of incomplete immunization was observed in Afar, Dire Dawa, Harari, Somalia, the western part of the SNNPR, and Gambella regions, ranging from 71 to 100%. On the other hand, in Addis Abeba, the central part of Oromia, the northern part of SNNPR, and some parts of the Amhara and Tigray regions, the level of complete immunization ranged from 0 to 26% (Figure 1). The dots in the figures represent the number of incompletely immunized children in each cluster, whereas, polygon are used to outline boundaries administrative regions in Ethiopia.

#### 3.4.1 Spatial and incremental autocorrelation result of incomplete immunization in Ethiopia

**Figure 2:**
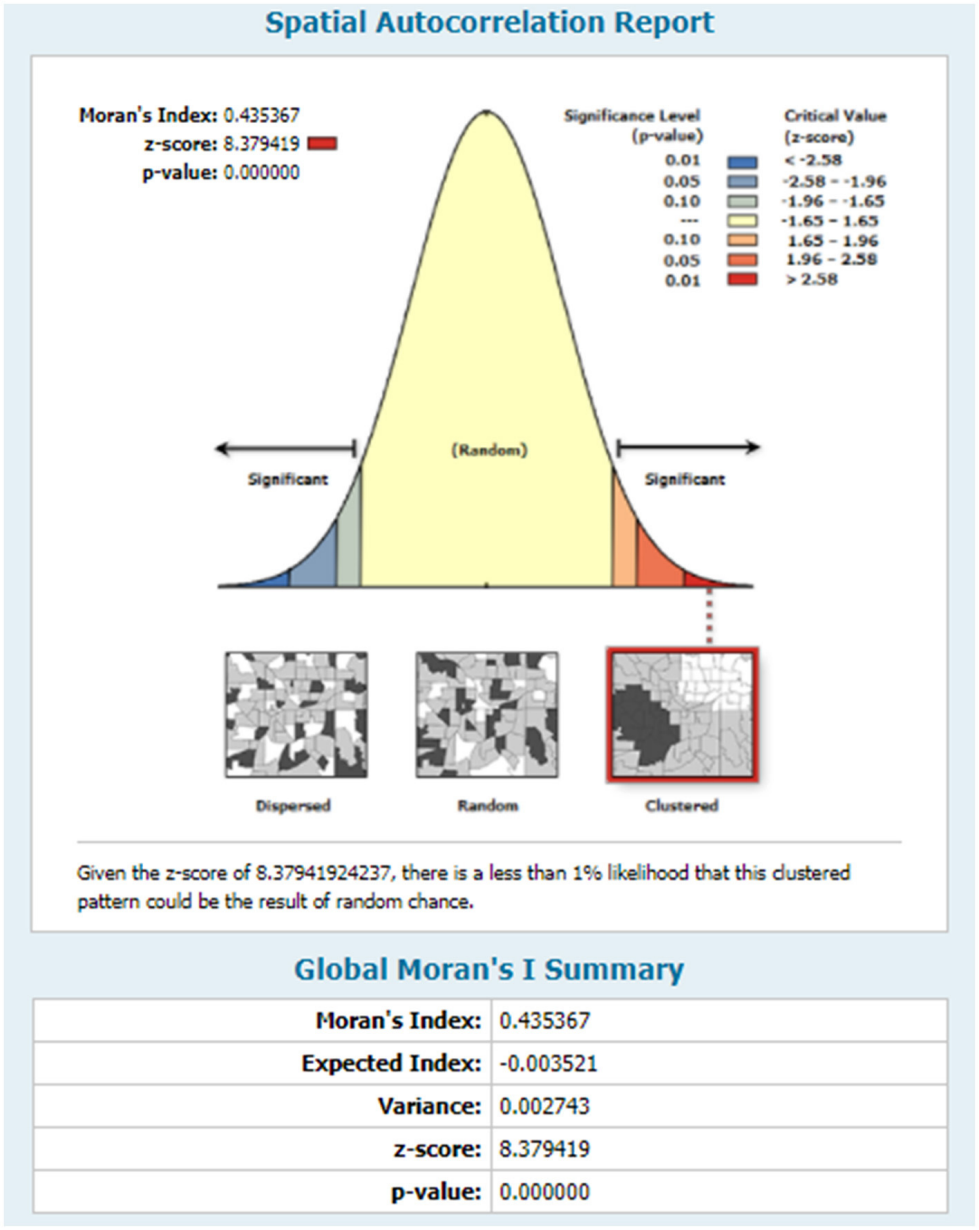
Spatial autocorrelation analysis status of incomplete Immunization in Ethiopia, using EDHS 2019 survey.

**Figure 3:**
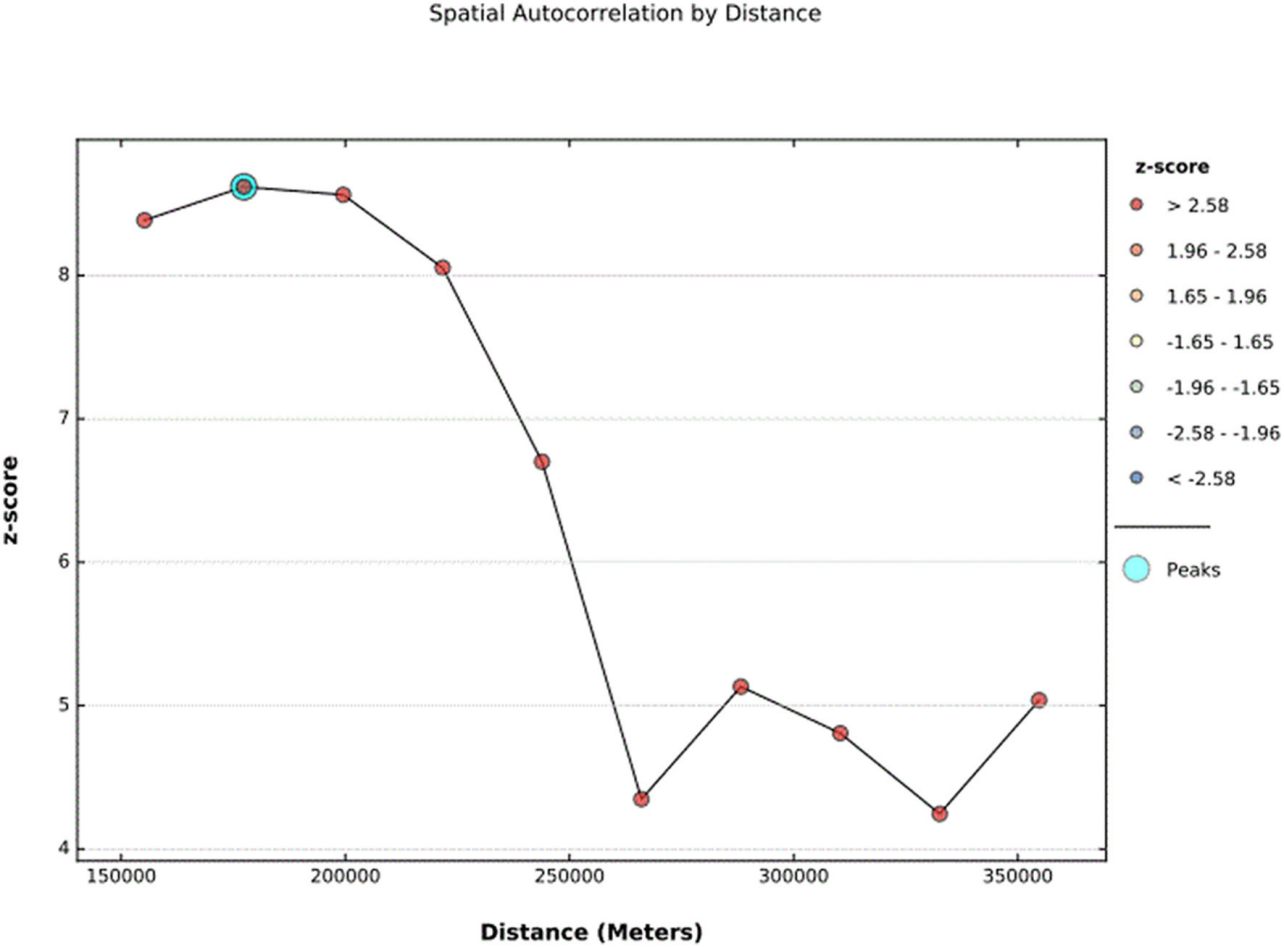
The spatial incremental autocorrelation analysis result of incomplete Immunization in Ethiopia using 2019 EDHS survey.

In the 2019 Mini-EDHS survey, incomplete immunization was not randomly distributed in Ethiopia. The global Moran's I value was 0.435367 (*Z*-score = 8.379419, *p*- value < 0.0.001), which indicates incomplete immunization is clustered in certain areas of Ethiopia The bright red and blue color's at the ends of the tails indicates an increased significance level in the distribution nature of incomplete Immunization (Figure 2). The incremental spatial autocorrelation for a series of distances presented by a line graph with a corresponding *z*-score was fitted to determine the average nearest neighbor and the maximum distance by which the spatial process promoting clusters is more pronounced. In line with this, a total of 10 distance bands were detected at a beginning distance of 155.19 kilometers (km). The *z*-score indicated that the first maximum distance at which the spatial clustering of incomplete immunization was most pronounced at 177.36 km (Figure 3).

#### 3.4.2 Hot-spot area analysis result of incomplete immunization in Ethiopia

**Figure 4:**
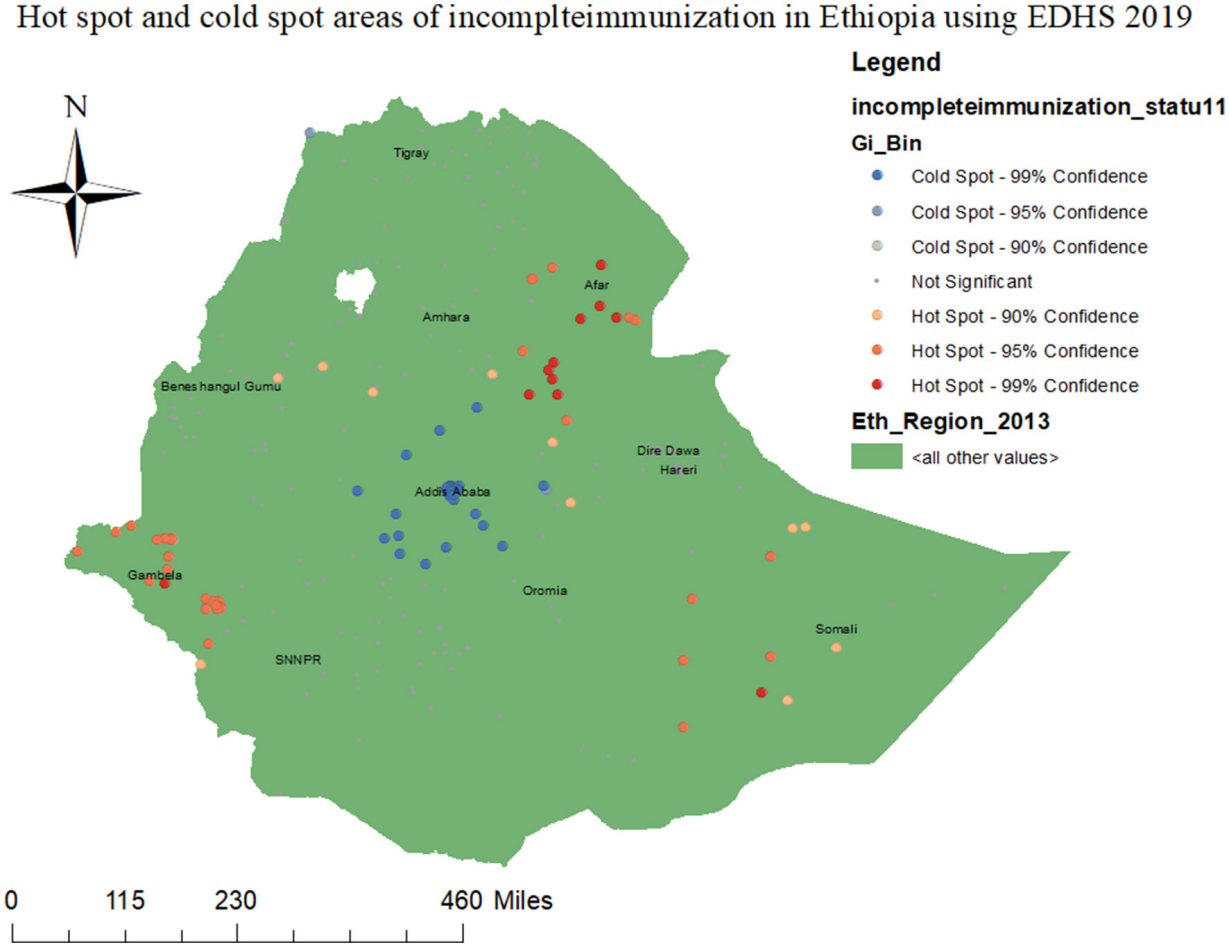
Hot spot and cold spot areas of incomplete immunization in Ethiopia using EDHS 2019 survey.

The Local Moran's *I* analysis results for incomplete immunization indicate that the distribution of incomplete immunization is not uniform across clusters in Ethiopia. As illustrated in the figure below, the red color indicates clusters with a high proportion (hot spot areas) of incomplete immunization, which were observed in the eastern part of Ethiopia (Afar, Somali, and Harar regions), Gambela, and the western part of the SNNPR region. On the other hand, cold spot areas of incomplete immunization are detected in central Ethiopia (Addis Ababa, the central part of Oromia, and the northern part of the SNNPR region) (Figure 4).

#### 3.4.3 The incremental interpolation result of incomplete immunization in Ethiopia

**Figure 5:**
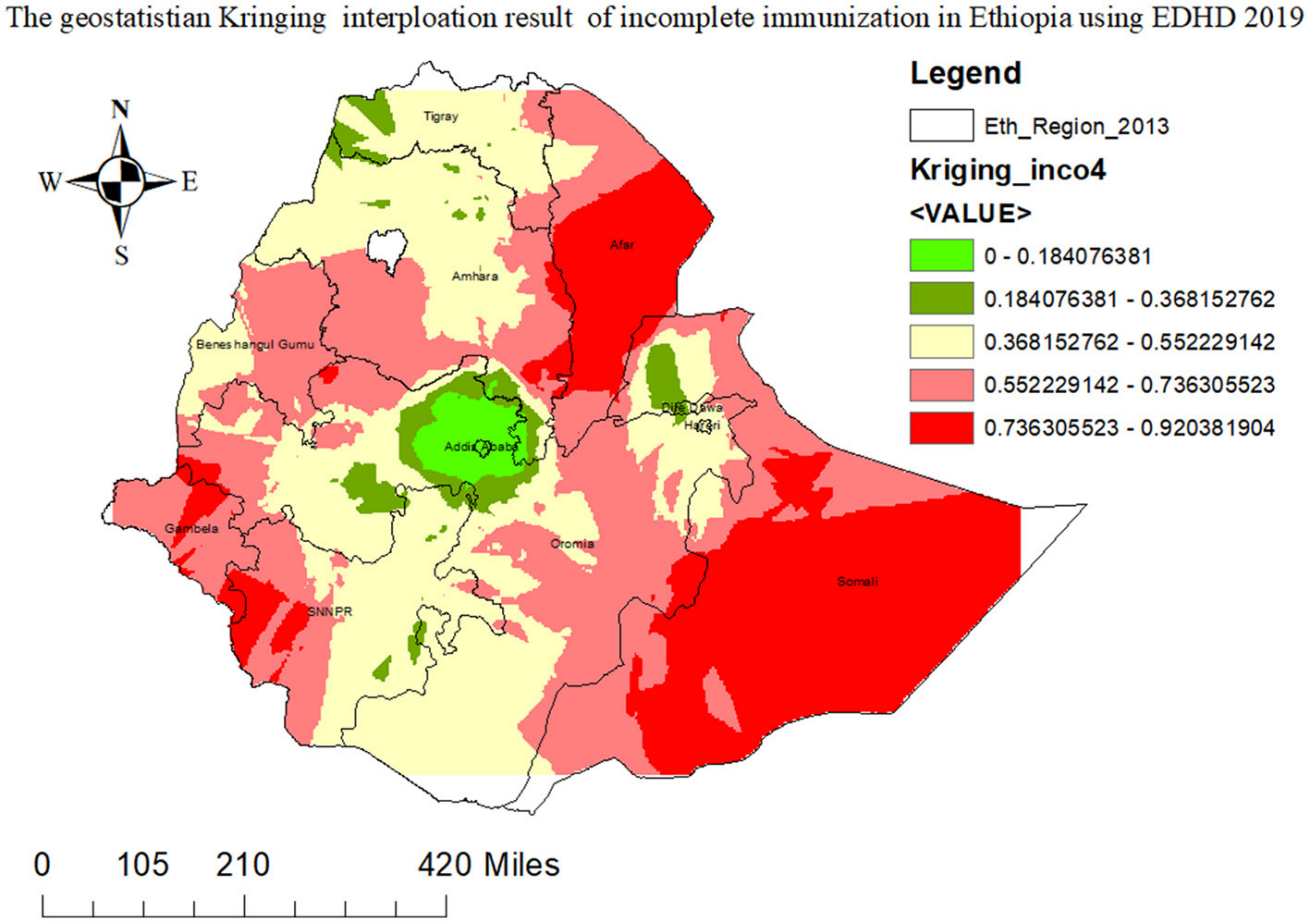
The geospatial kriging interpolation prediction graph of incomplete immunization in Ethiopia using 2019 EDHS survey.

In order to predict the burden of incomplete immunization in unsampled areas, we used the ordinary Kriging geo-reference statistical interpolation technique. Based on geo-statistical kriging analysis, in the 2019 mini-EDHS survey, the Somali region, Afar, Gambela, western SNNPR, western Amhara, and some parts of the Oromia region were the high-risk areas of incomplete immunization where by its prevalence ranges from 60.7154 to 94.989% (Figure 5).

#### 3.4.4 SatScan spatial analysis result of incomplete immunization in Ethiopia

**Figure 6:**
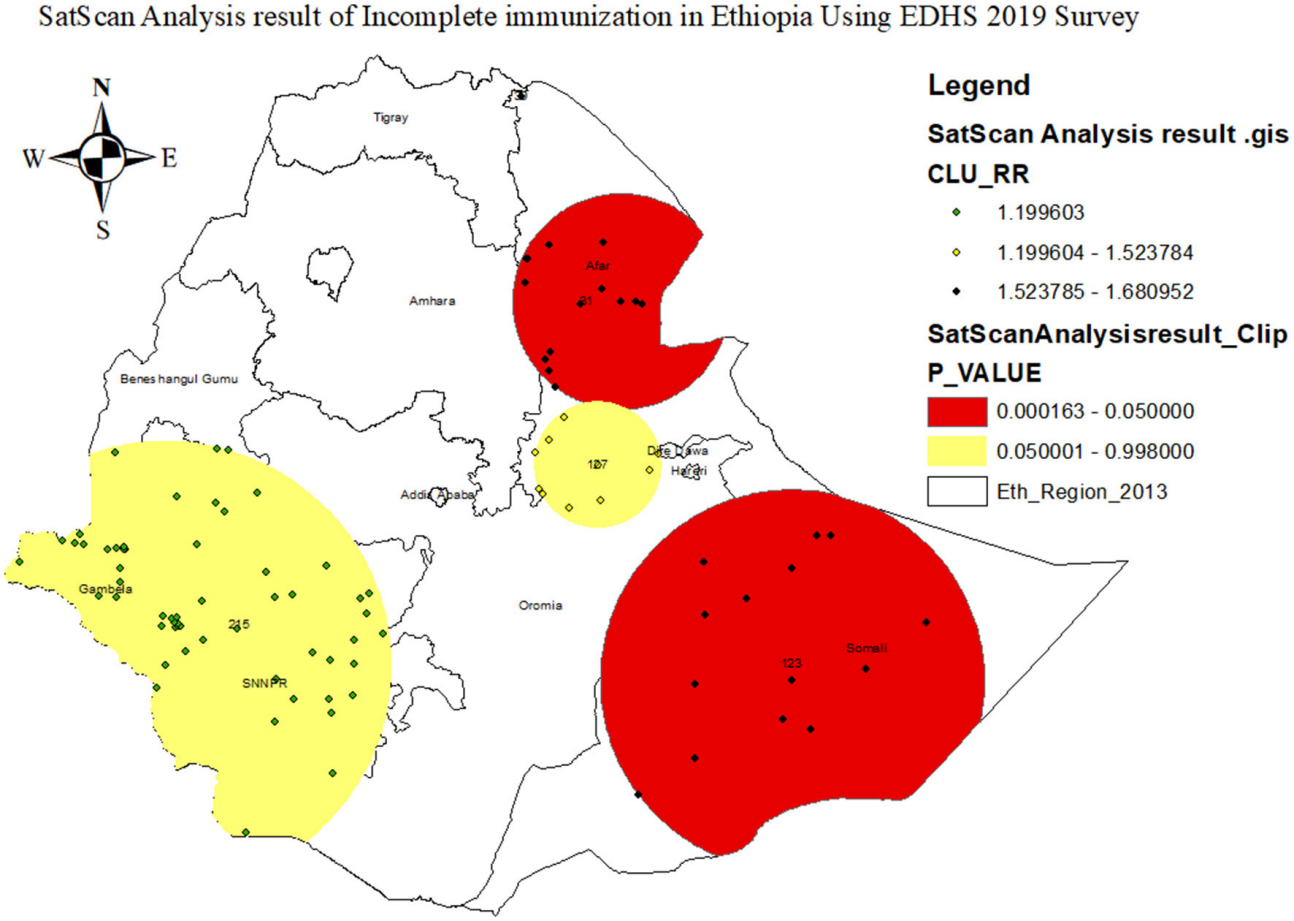
SaTScan hot spot analysis result of incomplete immunization in Ethiopia using 2019 EDHS survey.

As presented in Figure 6 and Table 4 below, the SaTScan spatial analysis detected a total of 55 statistically significant clusters of incomplete immunization status in Ethiopia. This implies that the burden of incomplete immunization was higher inside the SaTScan circular window compared to outside the SaTScan window. The most likely primary SaTScan cluster of incomplete immunization was detected in the Afar region, particularly in zones 1, 3, and 4 administrative zones (coordinates =11.561794 N, 41.244869 E, radius = 159.37 km, LLR = 14.803445, *p* < 0.001). Moreover, the most likely secondary SaTScan clusters were identified in Jarar, Doola, Korahe, Shabelle, Nogob, and Afdar administrative zones of the Somali region (coordinates = 6.505335 N, 43.486778 E, radius = 281.60 km, LLR = 11.579865, *p* < 0.01) (Figure 6; Table 3).

**Table 3:** The most likely SatScan clusters of incomplete immunization in Ethiopia using EDHS 2019 survey.

**Year**	**Cluster**	**Area of clusters**	**Coordinate/ radius**	**Popul ation**	**Case**	**RR**	**LLR**	***P*-value**
2019 mini EDHS	Primary clusters	31, 26, 32, 30, 33, 34, 47, 45, 48, 44, 49, 29, 46, 50	11.561794 N, 41.244869 E)/159.37 km	55	48	1.68	14.803445	< 0.01
	Secondary clusters	123, 138, 137, 135, 145, 136, 134, 131, 142, 140, 122, 133, 132, 141	6.505335 N, 43.486778 E)/281.60 km	52	44	1.62	11.579865	< 0.01

RR, relative risk; LLR, log-likelihood ratio.

**Table 4:** Multilevel multivariable mixed–effect analysis result of incomplete immunization in Ethiopia, using mini-EDHS 2019 survey.

**Community and individual level factors**	**Models**			
	**Null model AOR (95%CI)**	**Model I (AOR 95%CI)**	**Model II (AOR 95%CI)**	**Model III (AOR 95%CI)**
**Respondents age**				
		1		**1**
		0.90 (0.85–0.96)		**0.92 (0.86–0.98)** ^*^
**Educational status**				
Unable to read and write		1		**1**
Primary		0.26 (0.12–0.54)		**0.29 (0.14–0.59)** ^*^
Secondary		0.37 (0.12–1.11)		**0.27 (0.11–0.96)** ^*^
Tertiary and above		0.23(0.07–0.8)		**0.26 (0.08–0.88)** ^*^
**Household Poverty level**				
Poor		1		1
Middle		1.17 (0.56–2.44)		1.20 (0.56–2.56)
Rich		0.74 (0.35–1.58)		0.91 (0.37–2.23)
**ANC utilization status**				
Yes		1		1
No		0.80 (0.35–1.84)		0.79 (0.34–1.85)
**Place of delivery**				
Health institution		1		1
Home		2.65 (1.26–5.56)		**2.44 (1.15–5.16)** ^*^
**PNC visit**				
Yes		1		1
No		2.10 (1.07–4.13)		**2.70 (1.4–5.29)** ^*^
**Birth order**				
Increase by one unit		1.07 (0.87–1.29)		1.03 (0.85–1.25)
**Community educational status**				
High			1	1
Low			1.21(0.65–2.27)	0.83 (0.44–1.55)
**Poverty status**				
Poor			1	1
Rich			1.23 (0.64–2.37)	0.81 (0.42–1.56)
**Residence**				
Urban			1	
Rural			3.83 (1.78-8.2)	**3.11 (1.36–7.14)**
**Region**				
Urban setting			1	
Agriculture-based			1.88 (0.83–4.22)	1.57 (0.64–3.87)
Pastoralist			4.24 (1.88–9.53)	**3.41(1.29–9.00)** ^*^
**Measure of random effect (variation)**				
Variance	1.90269	1.794513	1.64648	1.46924
ICC (%)	37%	35.3%	32.5%	30.9%
PVC (%)	Reference	6.02%	15.6%	29.5%
MOR	3.47187	2.72	2.71828	2.71
**Model fitness**				
Log likelihood	−676.85425	−614.98252	−655.45829	−607.77422
Deviance	1,357.71	1,229.965	1,310.92	**1,215.48**
AIC	1,357.708	1,253.965	1,324.917	**1,249.548**

1–Reference category; AOR, adjusted odds ratio; CI, confidence interval, ^*^ and old letter - statistically significant variables in the full model. The bold value indicates the variables significantly associated with incomplete immunization in the null model.

### 3.5 Multilevel mixed-effect analysis result

#### 3.5.1 Random effect analysis result of incomplete immunization in Ethiopia

As shown in the table below (Table 3), the results of intra-class correlation (ICC) in the empty mode showed that 64.5% of the total variation in the burden of incomplete immunization was attributed by the difference in the community. Therefore, a multilevel fixed-effect regression model that handles the dependency of observations within a cluster was reasonable to be fitted. In the multilevel fixed-effect regression model, the full model (model III) containing both the individual and community level variables was chosen as the best-fit model because it has the lowest deviance and AIC values (deviance = 1,214, AIC = 1,248.489, *p*-value = 0.0001). The MOR in the full model showed that the odd of incomplete immunization was about 2.71 times higher among mothers from a community with a high proportion of incomplete immunization compared to a low proportion of incomplete immunization. The proportional change in variance (PCV) in the final model also indicates that about 29.5% of the variation in the proportion of incomplete immunization status was explained by both community- and individual-level variables (Table 4).

#### 3.5.2 Fixed-effect analysis result of incomplete immunization in Ethiopia

Variables with a *P*-value < 0.25 in the bivariable multilevel mixed effect model were fitted into the final multivariable model. Finally, the respondent's age, residence, region, educational status, place of delivery, and PNC utilization status were identified as significant predictors of incomplete immunization.

As the age of the mothers increases by 1 year, the odds of incomplete immunization decrease by 8% (AOR: 0.92; 95% CI, 0.86–0.98). In comparison to their counterparts, children's from rural area were 3.11 times at risk of (AOR: 3.11, 95% CI: 1.36–7.14) incomplete immunization. In addition, the odds of incomplete immunization are 3.41 times higher (AOR: 3.41, 95% CI: 1.29–9.00) among children from pastoralist communities than among children from urban settings. When compared with children delivered at a health institution, incomplete immunization is 2.44 (AOR: 2.44, 95% CI: 1.15–5.16) times more common among children delivered at home. Children who have PNC service are 2.7 times at risk of being incompletely immunized when compared with their counterparts (AOR: 2.70, 95% CI: 1.4–5.29) (Table 4).

## 4 Discussion

Incomplete immunization of children remains a significant public health concern, resulting in an increase in vaccine-preventable diseases and subsequently high infant mortality rates. Consequently, this study aims primarily to investigate the spatial variations and underlying factors associated with incomplete immunization in Ethiopia, utilizing nationally representative data. Significant hot-spot areas of incomplete immunization were observed in clusters located in the Afar region, the Somali region, and the southwest part of Ethiopia. Additionally, the most likely primary clusters were identified in the Afar and Somali regions of Ethiopia. These findings may be attributed to several factors, including the presence of hard-to-reach areas, the nomadic lifestyle of populations residing in the Somali and Afar regions, inadequate infrastructure, and insufficient education regarding the importance of immunization (34, 35). This further implies the need for improving access to immunization services; strengthen outreach programs and addressing specific challenges such as infrastructures in such high-risk areas. The above findings were also supported by previous spatial analysis studies conducted in Ethiopia using EDHS 2016 data (14, 20).

In EDHS 2019, we found a substantially high proportion of incomplete immunization (54%, 95%: CI 48–58%), which is too far from the 2030 global immunization program target. This study is comparable with prior studies conducted in different areas of Ethiopia (36, 37), in Saharan African migrant children's in Moroco (38), pooled Demographic Health survey reports of 25 Sub-Saharan African countries (39). Whereas, the above figure is higher than a study conducted in Ethiopia (15, 40–42), Togo (43), Nigeria (44), Indonesia (45), Pakistan (46), India (47), and Brazil (48). The possible explanation for the observed discrepancy might be due to the fact that the EDHS is a nation-wide survey that addresses the most hard-to-reach areas that the previous primary studies would not cover, the difference in the study period (this study uses data collected in 2019), the discrepancy in terms of variations of policies against immunization services between countries, and socio-cultural and socio-demographic differences across countries (11, 47). On the other hand, the proportion of incomplete immunization is lower than that found in a study conducted in the Somali region of Ethiopia (49). This could be attributed to the region's poor health infrastructure, leading to insufficient outreach programs and limited access to vaccines. Moreover, the nomadic lifestyle of residents presents challenges for vaccine accessibility through routine Expanded Program on Immunization (EPI) initiative (8, 35). Similarly, the mixed effect multilevel analysis revealed that the odd of incomplete immunization is 3.41 times higher in children from pastoralist communities (Somali, Afar, and Gambela) compared to children from urban settings (Addis Ababa and Dire Dawa regions. Both the spatial analysis and multilevel analysis findings suggest that outreach programs with mobile immunization sites and other innovative approaches need to be designed to address such hard-to-reach areas (23).

Socio-demographic characteristics, including the age of the mother, educational status, residence, and poverty level, were identified as significant predictors of incomplete immunization. With respect to this, as the age of the mother increases by 1 year, the odd of incomplete immunization of children decreases by 8%. This finding is in line with a previous study conducted in Arbegona district, southern Ethiopia (50) and Afghanistan (51). The possible elucidation for the observed association may be that older mothers tend to have more children, resulting in greater exposure to information and health education about the advantages of complete childhood immunization. This underscores the necessity of offering thorough health education and counseling to younger mothers.

In the current study, it is also observed that children's in rural areas were more than three times at risk for incomplete immunization compared to their counterparts. This is parallel with a study undertaken in in different setting of Ethiopia (15, 16, 49, 52–54), Kenya (55), Nigeria (56), Indonesia (45), and India (47). The possible explanation for the discrepancy could be due to the fact that children are in rural communities may not have good access to health services, good information and awareness on the most important diseases prevention and health promotion activities. Increased cost for transportation and low health seeking behavior in rural community will also be the other possible reasons on the high burden of incomplete immunization in these areas (57). This further implies that multi-sectorial collaboration and coordination will have needed to be strengthened to scale up access to health institutions, relieve transportation problems and other challenges in rural community.

Maternal educational status was another important predictor of incomplete immunization. In this aspect, the proportion of incomplete immunization significantly decreases as the educational status of the mother increases. This finding coincides with prior studies conducted in various areas in Ethiopia, Somalia, Kenya, Togo, and nine sub-Saharan African countries (41, 42, 52, 54, 55, 58, 59). This is due to the fact that mothers who do have good educational status will have better knowledge, attitudes, values, and information gathering skills about the benefits of full immunization in children. Thus, addressing disparities in educational attainment can contribute to higher immunization rates and better health outcomes for children.

Moreover, the current study also identified a significant association between the proportion of incomplete immunization and the routine utilization of maternal health services. For instance, children who were born outside health institutions were at the highest risk for incomplete immunization compared with their counterparts. This can be best explained by the fact that the Expanded Program on Immunization (EPI), both at national and international levels, highly recommends the integration of child health services (including immunization and postnatal care) with maternal health services (3, 60, 61). Hence, mothers who give birth at health institutions are more likely to receive sufficient counseling and education about the overall aspects of childhood immunization. This finding is in agreement with prior studies carried out in several areas of Ethiopia (18, 52, 62, 63), Somalia (59), Congo (64), Senegal (65), nine SSA countries (42), and Indonesia (66).

Finally, the odds of incomplete immunization are higher among mothers who didn't attend postnatal care (PNC) visits compared to mothers who had PNC visits. The observed association is in agreement with similar studies conducted in Ethiopia (15, 54, 67), Congo (64), India (68). This might be because providing appropriate counseling and support about childhood immunization is a main component of PNC services, indirectly increasing children's health-seeking behaviors and immunization coverage. Indeed, mothers would have more opportunities to receive messages on the benefits of childhood vaccination when they do have frequent PNC visits. This further suggests that improving maternal continuum of care is an essential strategy to scale up childhood immunization.

### 4.1 Strengths and limitations of study

The major strength of this study is the use of nationally representative data. Additionally, the utilization of the multilevel fixed-effect regression analysis method also plays an essential role in designing interventions targeting specific factors related to incomplete immunization.

On the other hand, this study had several limitations that need to be addressed by future researchers. First, since the study uses secondary data, we are unable to investigate the association of factors including the availability and accessibility of vaccines, the perceived fear of vaccine side effects, and the perceived vulnerability and severity of vaccine-preventable diseases with incomplete immunization. Second, given that the study is retrospective, recall bias might be introduced.

## 5 Conclusion and recommendation

The findings of this study conclude that the proportion of incomplete immunization in Ethiopia is significantly high, which deviates significantly from the WHO immunization program target. Additionally, incomplete immunization in Ethiopia was not randomly distributed and clustered in the Somali, Afar, and southwest regions of the country. Several factors, including mothers' socio-demographic status, residence, place of delivery, and utilization of postnatal care (PNC) services, significantly affect childhood immunization status in Ethiopia. Minimizing the variation in socio-demographic status through scaled-up multi-sectorial collaboration and strengthening maternal health care service utilization need to be considered by stakeholders.

## Data availability statement

All the data used to draw the conclusion of this study are available in the manuscript. There is also ethical restriction on sharing licensed codes or scripts for the current manuscript because the study used secondary data from the DHS database.

## Ethics statement

The studies involving humans were approved by the Demographic and Health Surveys (DHS) Program ICF 530 Gaither Road Suite 500 Rockville, MD 20850 USA. The studies were conducted in accordance with the local legislation and institutional requirements. Informed consent was not required because the study used secondary data (EDHS 2019 data). Instead, permission to get access to download DHS data was obtained from the major DHS data archivist through a reasonable request using the link http://www.dhsprogram.com. In the DHS data, there are no names of individuals or household addresses. The information retrieved was only used for statistical reporting and analysis of our registered research.

## Author contributions

BB: Writing—review & editing, Writing—original draft, Visualization, Validation, Supervision, Software, Resources, Project administration, Methodology, Investigation, Funding acquisition, Formal analysis, Data curation, Conceptualization. NA: Writing—review & editing, Validation, Supervision, Software, Project administration, Methodology, Formal analysis. GAd: Writing—review & editing, Validation, Supervision, Software, Project administration, Methodology, Formal analysis. GAm: Writing—review & editing, Validation, Supervision, Software, Project administration, Methodology, Formal analysis, Data curation. MM: Writing—review & editing, Supervision, Software, Project administration, Methodology, Formal analysis, Data curation. EChe: Writing—review & editing, Validation, Supervision, Software, Project administration, Methodology, Formal analysis, Data curation. MA: Writing—review & editing, Validation, Supervision, Software, Project administration, Methodology, Investigation, Formal analysis, Data curation, Conceptualization. MG: Writing—review & editing, Validation, Supervision, Software, Project administration, Methodology, Investigation, Formal analysis, Data curation, Conceptualization. KT: Writing—review & editing, Validation, Supervision, Software, Project administration, Methodology, Investigation, Formal analysis, Data curation, Conceptualization. MZ: Writing—review & editing, Supervision, Software, Methodology, Investigation, Data curation, Conceptualization. DA: Writing—review & editing, Visualization, Validation, Supervision, Software, Project administration, Methodology, Investigation, Formal analysis, Data curation, Conceptualization. AD: Writing—review & editing, Supervision, Software, Methodology, Investigation, Formal analysis, Data curation, Conceptualization. SF: Writing—review & editing, Visualization, Validation, Supervision, Software, Project administration, Methodology, Investigation, Formal analysis, Data curation, Conceptualization. TD: Writing—review & editing, Validation, Supervision, Software, Project administration, Methodology, Investigation, Formal analysis, Data curation, Conceptualization. ECha: Writing—review & editing, Visualization, Validation, Supervision, Software, Methodology, Investigation, Formal analysis, Data curation, Conceptualization. SK: Visualization, Writing—review & editing, Validation, Supervision, Software, Project administration, Methodology, Investigation, Formal analysis, Data curation, Conceptualization. WB: Writing—review & editing, Visualization, Validation, Supervision, Software, Project administration, Methodology, Investigation, Formal analysis, Data curation, Conceptualization. NM: Writing—review & editing, Visualization, Validation, Supervision, Software, Project administration, Methodology, Investigation, Formal analysis, Data curation, Conceptualization. YK: Writing—review & editing, Validation, Supervision, Software, Project administration, Methodology, Investigation, Formal analysis, Data curation, Conceptualization.
